# Deep Learning Analysis of Surgical Video Recordings to Assess Nontechnical Skills

**DOI:** 10.1001/jamanetworkopen.2024.22520

**Published:** 2024-07-31

**Authors:** Rayan Ebnali Harari, Roger D. Dias, Lauren R. Kennedy-Metz, Giovanna Varni, Matthew Gombolay, Steven Yule, Eduardo Salas, Marco A. Zenati

**Affiliations:** 1 Mass General Brigham, Harvard Medical School, Boston, Massachusetts; 2 Department of Psychology, Roanoke College, Salem, Virginia; 3 Department of Information Engineering and Computer Science, University of Trento, Trento, Italy; 4 School of Interactive Computing, Georgia Institute of Technology, Atlanta; 5 Department of Clinical Surgery, University of Edinburgh, Edinburgh, United Kingdom; 6 Department of Psychological Sciences, Rice University, Houston, Texas; 7 VA Boston Healthcare System, West Roxbury, Massachusetts

## Abstract

**Importance:**

Assessing nontechnical skills in operating rooms (ORs) is crucial for enhancing surgical performance and patient safety. However, automated and real-time evaluation of these skills remains challenging.

**Objective:**

To explore the feasibility of using motion features extracted from surgical video recordings to automatically assess nontechnical skills during cardiac surgical procedures.

**Design, Setting, and Participants:**

This cross-sectional study used video recordings of cardiac surgical procedures at a tertiary academic US hospital collected from January 2021 through May 2022. The OpenPose library was used to analyze videos to extract body pose estimations of team members and compute various team motion features. The Non-Technical Skills for Surgeons (NOTSS) assessment tool was employed for rating the OR team’s nontechnical skills by 3 expert raters.

**Main Outcomes and Measures:**

NOTSS overall score, with motion features extracted from surgical videos as measures.

**Results:**

A total of 30 complete cardiac surgery procedures were included: 26 (86.6%) were on-pump coronary artery bypass graft procedures and 4 (13.4%) were aortic valve replacement or repair procedures. All patients were male, and the mean (SD) age was 72 (6.3) years. All surgical teams were composed of 4 key roles (attending surgeon, attending anesthesiologist, primary perfusionist, and scrub nurse) with additional supporting roles. NOTSS scores correlated significantly with trajectory (*r* = 0.51, *P* = .005), acceleration (*r* = 0.48, *P* = .008), and entropy (*r* = −0.52, *P* = .004) of team displacement. Multiple linear regression, adjusted for patient factors, showed average team trajectory (adjusted *R^2^* = 0.335; coefficient, 10.51 [95% CI, 8.81-12.21]; *P* = .004) and team displacement entropy (adjusted *R^2^* = 0.304; coefficient, −12.64 [95% CI, −20.54 to −4.74]; *P* = .003) were associated with NOTSS scores.

**Conclusions and Relevance:**

This study suggests a significant link between OR team movements and nontechnical skills ratings by NOTSS during cardiac surgical procedures, suggesting automated surgical video analysis could enhance nontechnical skills assessment. Further investigation across different hospitals and specialties is necessary to validate these findings.

## Key Points

**Question:**

Is it feasible to automatically assess an operating room (OR) team’s nontechnical skills through deep learning–based analysis of surgical videos?

**Findings:**

This cross-sectional study of 30 cardiac surgical procedures found specific OR team members’ motion features, such as average trajectory and displacement acceleration, to positively correlate with higher nontechnical skills performance as assessed by the Non-Technical Skills for Surgeons (NOTSS) assessment tool, while displacement entropy was negatively correlated with NOTSS scores. These findings suggest that certain patterns of team motion in the OR are associated with a team’s nontechnical skills.

**Meaning:**

This study suggests the feasibility of applying deep learning methods to analyze surgical videos and assess an OR team’s nontechnical skills, which could be used for surgical education and quality improvement initiatives.

## Teaser

This cross-sectional study uses automated video analysis of cardiac surgery teams to examine operating room behaviors associated with higher nontechnical skill performance.

## Introduction

Nontechnical skills are critical for health care teams to operate successfully in high-stress and high-stakes environments, such as surgical settings, in order to deliver safe and effective care.^1,2^ Many incidents reported to the National Patient Safety Agency are attributed to poor nontechnical skills in surgical teams.^3,4^ Nontechnical skills encompass communication, teamwork, leadership, situational awareness, and decision-making, all of which have significant impact on technical skills and overall individual and team performance in the operating room (OR).^5,6^

Several efforts have been made to develop assessment tools to train and evaluate nontechnical skills proficiency of clinicians. These tools were designed to evaluate the skills of individual surgical team members, such as Non-Technical Skills for Surgeons (NOTSS) for attending surgeons,^7^ Anesthetists’ Nontechnical Skills (ANTS) for anesthesiologists,^8^ Perfusionists’ Intraoperative Non-Technical Skills (PINTS) for perfusionists,^9^ and Scrub Practitioner’s List of Intraoperative Nontechnical Skills (SPLINTS) for scrub nurses,^10^ as well as the overall teamwork of the surgical team (eg, NOTSS).^11^

While these assessment tools remain crucial for evaluating nontechnical skills in surgical settings, their reliance on subjective evaluation introduces significant methodological limitations. Primarily, the assessment of team processes—predicated on interaction, collaboration, and exchange—is largely dependent on retrospective subjective accounts by team members. Such reports are susceptible to biases and necessitate interruptions in ongoing activities for reflection. This highlights the need for new measurement tools capable of directly capturing process behaviors as they unfold. Moreover, such tools require a high level of expertise and skill from trained observers to reliably rate and evaluate nontechnical skills.^12,13^ Annotations can be a time-intensive process, requiring extensive training and evaluation periods, which limits scalability and increases cost. Finally, understanding team dynamics and the emergence of collective phenomena demands recognition of their temporal evolution.^14^ However, much of the research in this domain has employed static, cross-sectional designs, which fall short in capturing the fluidity and emergence of team processes over time.^15,16^ Consequently, a shift toward automated and real-time research methods is imperative to adequately reflect the nuanced and dynamic nature of team processes and effectiveness in surgical environments.^17,18,19^

Evidence from sports science and team analysis^11^ shows the significant association between team members’ physical movements and overall team performance,^20,21^ including aspects of communication, coordination, and situational awareness. For instance, motion analysis in sport science has been effectively used to predict team success, highlighting patterns of movement that correlate with strategic teamwork and performance outcomes.^22,23,24^ Similarly, in organizational studies, the analysis of physical dynamics within groups has offered insights into the efficiency of information flow and decision-making processes, underpinning the importance of nonverbal communication in team effectiveness. Despite the proven utility of motion metrics in these fields,^25,26,27,28^ the application of similar methodologies to assess nontechnical skills in surgical settings remains underexplored. This gap presents a unique opportunity to leverage motion analysis to enhance our understanding and evaluation of surgical teams. Specifically, we propose the use of deep learning (DL)-based pose estimation to automatically extract motion features from video recordings of surgical teams in action. Pose estimation techniques have shown promise in various domains for their ability to analyze complex visual data and extract meaningful patterns related to human behavior and interactions.^25,26,27,28^

By applying this approach, we aim to objectively quantify the physical movements of surgical team members and investigate whether and to which extent it can be related to expert-rated NOTSS scores. We hypothesize that the motion features extracted using OpenPose in surgical teams will have association with NOTSS scores, which served as the ground truth for surgical team nontechnical skills assessment. This study contributes to the development of a complementary tool to supplement existing assessment techniques in surgical nontechnical skills.

## Methods

### Study Design

**Figure 1.**
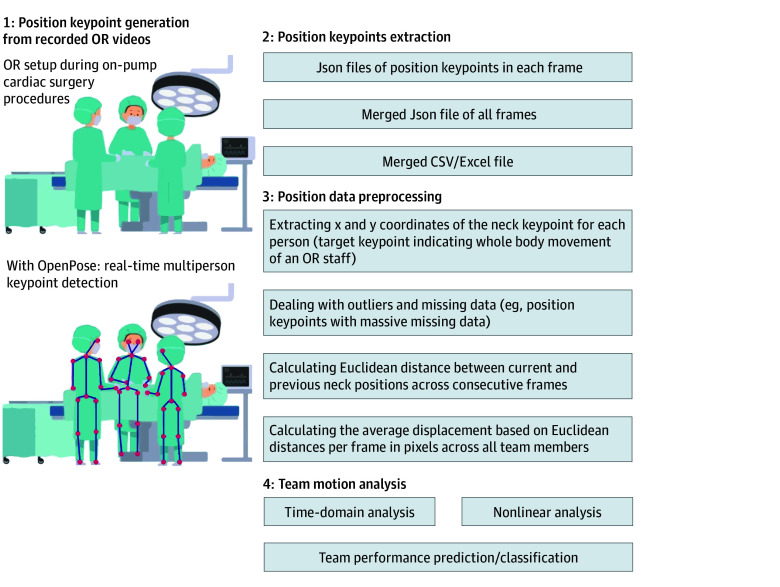
Methodology Overview for Operating Room (OR) Video Recording and Keypoint Processing

This study aimed to investigate the association of team motion features captured from pose estimation algorithms with nontechnical skills performance measured using the NOTSS tool. Data was prospectively collected from January 2021 through May 2022, focusing on a single critical phase of cardiac surgery: separation from bypass in which NOTSS scores on team-level and video-based motion features were extracted (Figure 1). This study followed the Strengthening the Reporting of Observational Studies in Epidemiology (STROBE) reporting guideline.

### Setting

The research was conducted within the cardiovascular OR of a tertiary teaching hospital. Audio-visual recordings of the surgical teams were collected using a customized portable observation lab (Mangold International GmbH), which included 4 lapel microphones and a wall-mounted high definition, 1920 × 1080p Axis P1375 RGB camera (Axis Communications) capturing wide fields of view of the OR at 30 frames per second.

### Participants

A cardiovascular OR team at a teaching hospital is typically composed of 4 subteams: (1) a cardiac surgical team consisting of an attending surgeon, one resident or fellow, and a surgical advanced practice clinician (ie, a physician assistant or nurse practitioner); (2) an anesthesiology team consisting of an attending anesthesiologist, 1 or more residents and/or fellows, and a nurse anesthetist; (3) a perfusion team consisting of a lead and an assistant perfusionist; and (4) a nursing team consisting of scrub nurses and circulating nurses. All surgical staff met inclusion criteria if they were between ages 18 and 70 years working in the OR as part of their usual duties, providing care for cardiac surgery patients in a tertiary academic hospital in the area of Boston, Massachusetts, and willing and able to provide informed consent for the study. The OR team participants were informed that their intraoperative motions and teamwork behaviors were being assessed (details in eMethods of Supplement 1). Patients met the inclusion criteria if they were over 18 years of age presenting for elective or urgent cardiac surgery isolated aortic valve replacement or repair (AVR), isolated coronary artery bypass graft (CABG), or combined AVR and CABG procedures as part of usual care and were willing and able to provide informed consent for the study. All patients provided written informed consent. The research protocol was approved by the institutional review boards at participating sites (with board names withheld to protect sensitive patient data). Data were collected from January 2021 through May 2022.

### Variables

#### NOTSS Scores

Three trained raters in the NOTSS behavioral rating system assessed overall teams’ nontechnical skills along 12 items (behavioral elements) grouped in the following 4 categories: situational awareness, decision-making, communication and teamwork, and leadership.^29^ Each of these raters had previously undergone standardized training to become familiar in NOTSS ratings. Each item was rated using a 4-item Likert scale (scored on a 4-point scale, with 1 indicating poor performance and 4, good performance). Given previous efforts demonstrating the acceptability of applying the NOTSS framework to the entire surgical team,^11^ assessments were made at the team level, focusing on the collective performance of the entire surgical team. The process involved retrospective Zoom sessions to watch surgical videos for NOTSS evaluations, with data recorded in REDCap. For each surgical case, NOTSS scores were averaged across the raters.

### Motion Features

#### Motion Data Extraction

**Figure 2.**
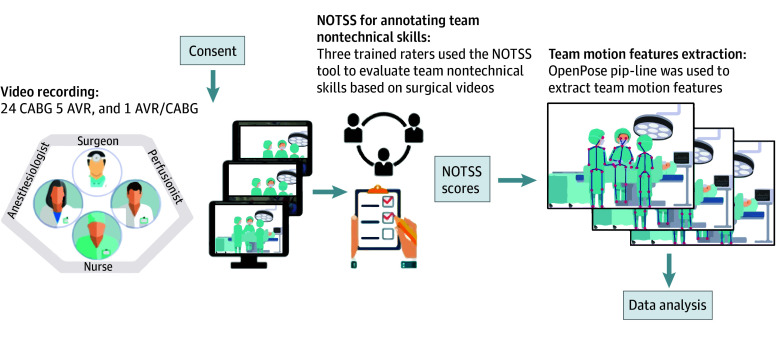
Pipeline of Surgical Team Analysis Generation of position keypoints with OpenPose and data processing are described in Figure 1. Aggregating data from all frames created a final file containing the x and y locations of keypoints of each OR team member in each time frame. According to our previous studies^29^, we suggest using only the neck keypoints because changing the coordination of this keypoint resulted in higher accuracy in capturing the whole-body movements of a person. After dealing with outliers and missing data, the x and y coordinates of the neck keypoint were used to calculate the Euclidean distance between current and previous neck positions across consecutive frames. The average displacement per frame in pixels then was calculated across all team members to capture the entire team motion. Team displacement data measured were analyzed using time-domain and nonlinear methods. AVR indicates aortic valve replacement or repair; CABG, coronary artery bypass graft; NOTSS, Non-Technical Skills for Surgeons; OR, operating room.

The OpenPose library, a real-time multiperson keypoint detection library for pose estimation,^30^ was used to extract the 2-dimensional body poses of each member of the OR teams. Each body pose consists of 17 keypoints, as depicted by the colored wire-frame figures in Figure 2. Although OpenPose is not specifically designed for the OR, it was deemed appropriate for analysis in the OR due to its ability to estimate multiperson body poses in complex and dynamic environments.^31^ Our focus was on team dynamics and collective motions of individuals, rather than individual motion. Therefore, the use of this approach allowed us to compute teams’ motion features, which was essential to achieving our research objectives.

#### Motion Feature Selection

Our selection of motion features for analysis was informed by a review of existing research on team dynamics across various fields, including sports science and organizational psychology. Team displacement and trajectory were associated with team coordination and task allocation during high-stakes resuscitation,^32^ mass casualty incidents,^33^ team-based sports,^22,23,24^ and organizational psychology for nonverbal synchrony.^34^ Similarly, these metrics have been used in sports analysis to evaluate dynamic interpersonal coordination among players, as well as to gauge team communication through speed, speed variability, and acceleration.^22,23,35^ Displacement entropy also have been used to capture variability and irregularity in displacement data, and it was correlated with teamwork in health care.^36,37^ Formulas for motion features used for analysis are provided in eTable 1 of Supplement 1 (data preprocessing available in eMethods of Supplement 1).

Motion features included displacement, trajectory, speed, speed variability, acceleration, and entropy. The displacement of each person in the team was calculated as the Euclidean distance between their current and previous neck positions across consecutive frames. Trajectory was calculated by summing the displacements over time for each team member.

 The speed of each team member was calculated by computing neck displacement between consecutive frames and dividing by the time between frames. This results in a measure of how fast each person was moving at each point in time. To calculate speed variability, the standard deviation of individual speeds within the team was computed at each time point. Speed variability measured the fluctuation or variation in the speed of team member over time. The acceleration of each team member was calculated as the difference in speed between consecutive frames. This results in a measure of how fast each team member was accelerating or decelerating at each point in time. Shannon entropy^38^ was calculated by transforming the continuous team displacement data into a symbolic sequence using nonoverlapping 60-second time window. This involved categorizing displacement into distinct symbols, each representing a specific movement pattern. Higher entropy indicates more erratic movements, while lower values suggest uniform, predictable patterns.^39^

### Statistical Analysis

The Kolmogorov-Smirnov test indicated a nonnormal distribution of these features. Interrater reliability (IRR) analysis for the NOTSS rating was performed using the mean measures intraclass correlation estimated by an α 2-way mixed model with consistency type. The intraclass correlation coefficient (ICC) was reported with a 95% CI. We performed correlation analysis to investigate the association between NOTSS scores and motion features using a significance threshold of *P* < .05. Those variables in motion features deemed to be significant were further analyzed through multiple linear regression adjusted for preoperative (30-day morbidity) and intraoperative (bypass length) factors. Analyses were performed using Python version 3.2 (Python Software Foundation) for data extraction and preprocessing and SAS version 9.3 (SAS Institute) for statistical analysis.

## Results

### Participants

#### Patients

The dataset included 30 complete cardiac surgery procedures: 26 (86.6%) were on-pump CABG and 4 (13.4%) were AVR (Table). All patients were male, with a mean (SD) age of 72 (6.3) years.

**Table.** Patient Demographic and Procedure Characteristics

Variables	Measures, mean (SD)
Male sex, No. (%)	30 (100)
Age, y	72.0 (66.0-75.0)
BMI	29.6 (27.4-32.5)
BSA, m^2^	2.1 (1.9-2.2)
Preoperative risk (VASQIP risk assessment core)	
30-d morbidity risk, %	6.9 (6.7-9.6)
30-d SSI risk, %	1.2 (0.9-2.0)
**Surgical process and outcome measures**	
Surgery duration, min	329.0 (266.0-371.0)
Bypass duration, min	112.0 (94.0-134.0)
Hospital length of stay, d	7.0 (7.0-9.0)
Mortality, No. (%)	0

Abbreviations: BMI, body mass index (calculated as weight in kilograms divided by height in meters squared); BSA, body surface area; VASQIP, Veteran Affair Surgical Quality Improvement Program; SSI, surgical site infection.

#### Teams

Our study involved 30 surgical teams, comprising 26 unique individuals across 4 roles: 5 anesthesia providers, 5 perfusionists, 3 surgeons, and 13 nurses. Each of the 30 teams was configured with 1 member from each of these roles. All surgical teams were composed of 4 key roles (attending surgeon, attending anesthesiologist, primary perfusionist, and scrub nurse) with additional supporting roles.

### NOTSS Scores

Across the 30 cases, the mean duration of the separation from the bypass phase was 7.8 (95% CI, 3.7-10.3) minutes. Mean NOTSS scores were 3.3 (95% CI, 3.0-3.5) for situational awareness, 3.2 (95% CI, 3.0-3.5) for decision-making, 3.3 (95% CI, 3.0-3.5) for communication and teamwork, and 3.2 (95% CI, 3.0-3.3) for leadership. The IRR analysis conducted with 3 raters yielded an ICC of 0.070 (95% CI, −0.110 to 0.225; *P* = .21). Upon conducting the IRR analysis for 2 raters, the results indicated an improvement in the ICC values. The ICC for average measures was 0.339 (95% CI, 0.187 to 0.463; *P* < .001). The overall NOTSS score was positively correlated with the duration of the separation from the bypass phase (ρ = 0.47, *P* = .008), meaning that teams that took longer to terminate the patient from bypass were rated as presenting better nontechnical skills.

### Motion Features

**Figure 3.**
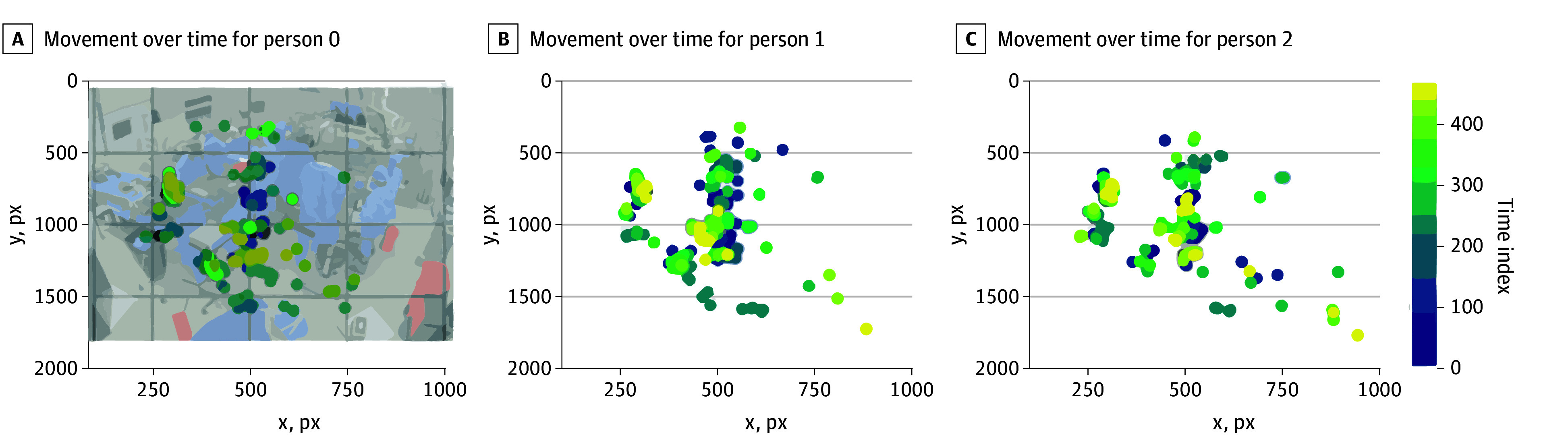
Movement Visualization of Surgical Team Members

Figure 3 presents a visualization of individuals and team movement dynamics. The color gradient illustrates the average temporal progression of individual movement team members within the OR throughout procedures.

### Correlation Analysis

**Figure 4.**
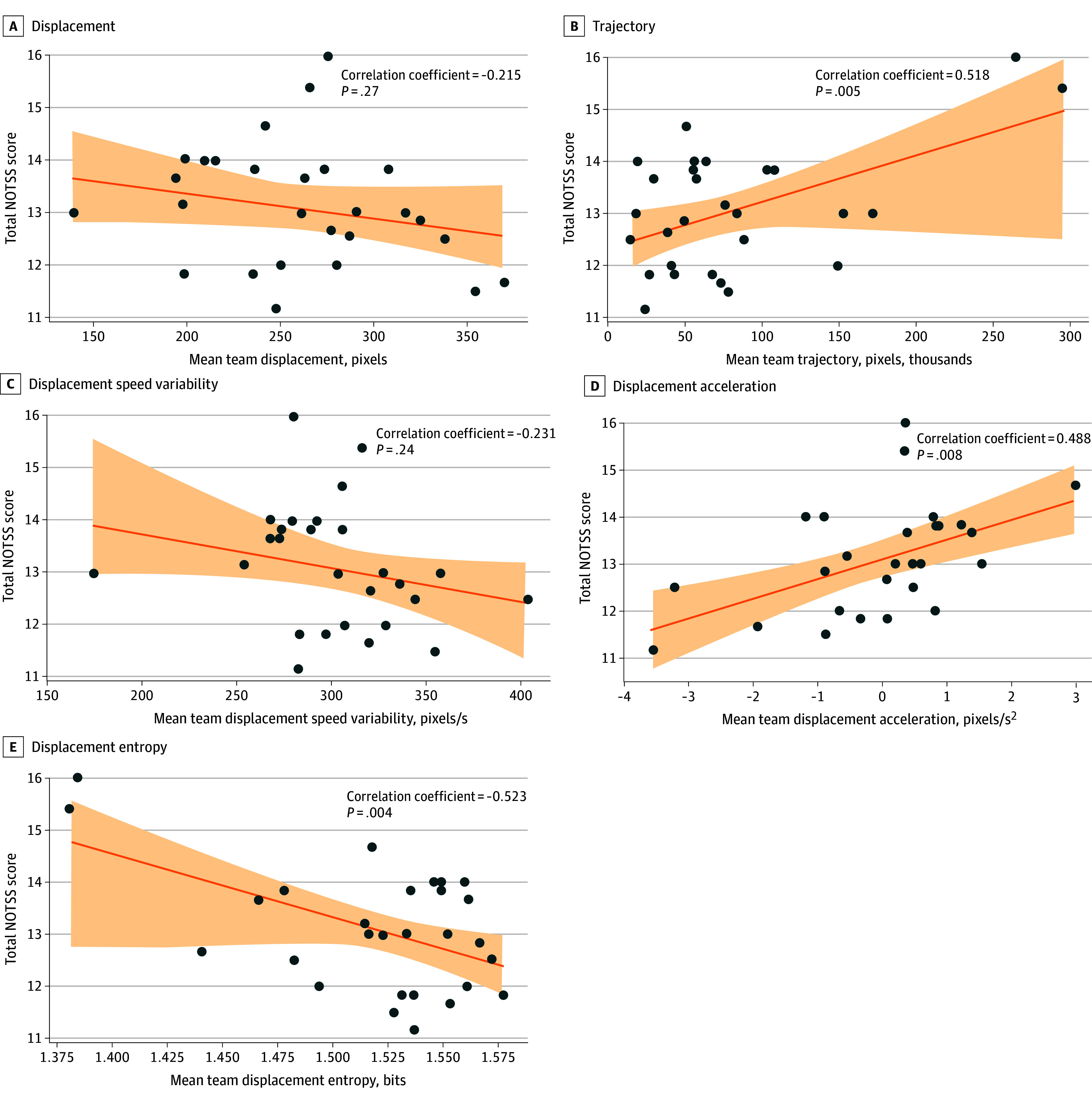
Correlation Analyses Between Total NOTSS Scores and Team Motion Features

In examining the association of team dynamics with nontechnical skill performance, significant correlations were found with certain movement features (Figure 4). The average trajectory and acceleration of team displacement showed a positive correlation with overall NOTSS scores (trajectory: *r* = 0.51; *P* = .005; acceleration: *r* = 0.48; *P* = .008). In contrast, team displacement entropy was inversely related to NOTSS scores, with a correlation coefficient of *r* = −0.52 (*P* = .004). There was no significant association observed between average team displacement (*r* = −0.21; *P* = .27) and speed variability (*r* = −0.23; *P* = .23) with NOTSS scores. Correlation of motion features with individual dimensions of NOTSS is provided in eFigure 1 in Supplement 1.

### Regression Analysis

Based on correlation analyses that identified significant associations between specific team motion features and total NOTSS scores, we performed a multiple linear regression analysis. These models examine the association between team motion features—trajectory, acceleration, and entropy of team displacement—and total NOTSS scores, adjusted for patient-related variables that include bypass length and 30-day morbidity (eTable 2 in Supplement 1). Results indicate significant models across all features with adjusted *R^2^* values ranging from 0.261 to 0.335, reflecting moderate explanatory power. The average team trajectory and team displacement acceleration models demonstrate positive associations with NOTSS scores, while the average team displacement entropy model exhibits a negative association. Detailed results of regression analysis are provided in eTable 2 in Supplement 1.

## Discussion

We investigated the feasibility of using computer vision-based body pose estimators of surgical team members to evaluate nontechnical skills during cardiac surgery based on motion features extracted from surgical video recordings. We aimed to explore the potential association between these team motion features and team nontechnical skills performance measured by NOTSS scores. Our results show that trajectory and acceleration of team displacement were positively correlated and entropy of team displacement was negatively correlated with total NOTSS scores. To our knowledge, these findings provide potentially useful insights into the association between motion performed by the members of surgical teams and nontechnical skills performance for the first time.

Bernieri and Rosenthal^40^ define team coordination as the presence of nonrandom, patterned behaviors in interactions. The findings of our study regarding displacement entropy support this notion: teams with higher NOTSS scores tend to show less entropy in their displacement.^39^ Research shows that less erratic and more anticipatory movements are characteristic of experienced surgical teams who possess knowledge of the task and the ability to predict their team partner’s actions.^41,42^ Moreover, similar observations have been made in team-based sports.^43,44^

A higher average trajectory of team displacement in our study, which measures collective distance traveled by all team members in the OR, may reflect a range of factors from increased physical activity to strategic spatial navigation within the OR. While positively correlated with higher NOTSS scores, it prompts consideration of how coordinated and purposeful movements contribute to effective team performance. Such coordination likely mirrors the team’s ability to respond to surgical demands efficiently, aligning with the principles of effective surgical teamwork.^29,45^ However, it is crucial to interpret these findings with caution because of the exploratory nature of our study.

Similarly, the positive correlation between higher displacement acceleration and NOTSS scores may also suggest that teams with the ability to swiftly adjust their movement speed in response to surgical demands tend to have better nontechnical skills. This adaptability likely reflects the team’s ability to respond effectively to the dynamic environment of the OR, showing their agility and collective efficiency. While we do not have direct evidence from the OR context, parallels can be drawn from sports science, where higher acceleration has been associated with improved teamwork, particularly in situational awareness.^35,46,47^ This concept aligns with the notion that team members can adapt their actions rapidly to meet the objectives of the task at hand, often without explicit consideration for what may be considered the optimal course of action. This evidence suggests that such behavior is in line with principles of naturalistic decision-making, where the success of such spontaneous decision-making is linked to an individual’s or team’s situational awareness.

### Limitations

This study had several limitations. While OpenPose software analyzed the entire surgical procedure, the NOTSS rating for the team was based on a relatively short segment of the procedure (ie, weaning from cardiopulmonary bypass). In addition, we recognize that the low IRR between 3 raters poses a significant limitation of our study. The variability in outcomes was partly due to the diverse training backgrounds of the 3 raters, which may have contributed to the observed discrepancies in ratings. Additionally, the use of the NOTSS scale, which operates on a constricted scale from 1 to 4, could have compounded the rating inconsistencies. However, a subsequent IRR analysis with 2 raters showed an improved consistency in the ICC values, suggesting that the number of raters and their homogeneity in background could be key factors in achieving reliable assessments.

The workflow within surgical teams typically involves scheduled breaks and shift changes, leading to planned substitutions of team members. Therefore, the motion data captured by this analysis may represent different individuals at various times, rather than a consistent set of team members throughout the entire procedure. This variability must be considered when correlating motion data with NOTSS scores, as the metrics may reflect the actions of a composite team rather than a single, unchanging unit. The small sample size also limits the generalizability of the results to a larger population of surgical teams. Additionally, our study focused exclusively on team performance during open cardiac surgery, which may limit the applicability of the findings to other surgical specialties and procedures. Future research should include larger sample sizes and diverse surgical settings to provide validity evidence to these preliminary findings and explore the potential applications of motion features extracted from computer vision-based algorithms in assessing nontechnical skills across various surgical domains.

## Conclusions

This study investigated the association between motion features extracted using computer vision-based pose estimator algorithms from cardiac surgery video recordings and NOTSS scores, the criterion standard for surgical nontechnical skills assessment. The findings showed novel associations between specific motion features and NOTSS scores in surgical teams. The integration of motion features analysis into nontechnical skills assessment tools offers a potentially groundbreaking approach for objective evaluation and feedback in surgical skills development and merits further studies.

## Notes

Supplement 1.
eMethods.
**eTable 1.** Formulas Used for Calculating Motion Features for Surgical Team **eFigure 1.** Movement Visualization of Surgical Team Members **eFigure 2.** Correlation Between Team Motion Features and NOTSS Scores **eTable 2.** Linear Regression Models Adjusted for Preoperative and Intraoperative Variables

Supplement 2.
Data Sharing Statement
