# Supplementary material for: Deep Learning Analysis of Surgical Video Recordings to Assess Nontechnical Skills
Source: JAMA Netw Open. 2024 Jul 31;7(7):e2422520. doi: 10.1001/jamanetworkopen.2024.22520 (PMC11292454; doi:10.1001/jamanetworkopen.2024.22520)
Supplement: Supplement 1. — eMethods. eTable 1. Formulas Used for Calculating Motion Features for Surgical Team eFigure 1. Movement Visualization of Surgical Team Members eFigure 2. Correlation Between Team Motion Features and NOTSS Scores eTable 2. Linear Regression Models Adjusted for Preoperative and Intraoperative Variables [file jamanetwopen-e2422520-s001.pdf]

Supplemental Online Content

Harari RE, Dias RD, Kennedy-Metz LR, et al. Deep learning analysis of surgical video recordings to assess nontechnical skills. *JAMA Netw Open.* 2024;7(7):e2422520. doi:10.1001/jamanetworkopen.2024.22520

eMethods.

eTable 1. Formulas Used for Calculating Motion Features for Surgical Team

eFigure 1. Movement Visualization of Surgical Team Members

eFigure 2. Correlation Between Team Motion Features and NOTSS Scores

eTable 2. Linear Regression Models Adjusted for Preoperative and Intraoperative Variables

This supplemental material has been provided by the authors to give readers additional information about their work.

© 2024 Harari RE et al. *JAMA Network Open.*

eMethods.

1. **OR Team Consent Process:**

Eligible hospital staff provided an informed consent, which was distinct from that completed by patients, prior to beginning any A/V recordings in a case that they were assigned to (i.e., any staff member who had any reason to enter the OR while the recording was in progress). Research staff obtained their informed consent at one time point, which encompassed any subsequent cases that clinical staff may have been involved in. To obtain consent, research staff hosted meetings with each specialty, during which time they presented relevant background material, described the procedures that would be involved (e.g., setting up cameras and microphones, asking representative members to equip themselves with the ECG device, overviewing self-report surveys they would be asked to respond to, etc.), and welcomed questions from the providers. At the end of these meetings, research staff collected informed consent documents from all providers present. The informed consent documents outlined that researchers were interested in observing teamwork indicators, including communication patterns, leadership style, behaviors, etc. Given that an earlier grant had funded a very similar, multi-year project involving many of the same research staff and providers, providers had already been exposed to the premise of this work for an extended period of time, diminishing the likelihood of the Hawthorne Effect persisting throughout the data collection period.

2. **Motion Features Formula:**
3. **Motion Data Pre-processing:**

To deal with possible noise in the extracted data, we downsampled data into 1-second time window and applied a two-stage filtering process to ensure the integrity of the signal. Initially, a low-pass Butterworth filter was employed to attenuate high-frequency noise, which is common in motion capture data [39,40]. This filter was chosen for its flat frequency response in the passband, minimizing the distortion of the signal's amplitude. Subsequently, a Savitzky-Golay filter was utilized to smooth the data, which preserves the signal's higher momenta and is particularly effective in reducing noise when computing derivatives[40–42].

© 2024 Harari RE et al. *JAMA Network Open.*

4. Movement Visualization

eFigure 1. presents a sample of team movement visualizations including average displacement and acceleration, and entropy over time.

5. Correlation Between Team Motion Features and Individual NOTSS Scores:

eFigure 2. presents a series of correlation analyses between four team motion features (Average Displacement, Average Trajectory, Speed Variability, and Displacement Entropy) and four NOTSS (Non-Technical Skills for Surgeons) categories (Decision Making, Situational Awareness, Communication & Teamwork, and Leadership). Each subplot displays a scatter plot with a linear regression line, along with the correlation coefficient and p-value for each analysis. The results show varying degrees of correlation across different motion features and NOTSS categories, with some combinations showing stronger relationships than others. Notably, Displacement Entropy demonstrates a significant negative correlation with Situational Awareness (r= -0.44) and Communication & Teamwork (r= -0.42) scores, suggesting that higher irregularity in displacement pattern in team movement may be associated with lower performance in these areas. Conversely, Avg. Team Trajectory shows positive correlation with Situational Awareness (r= 0.35) and Communication & Teamwork (r= 0.45) scores. These findings highlight the potential of motion analysis as a tool for assessing NTS in surgical environments.

© 2024 Harari RE et al. *JAMA Network Open.*

eTable 1. Formulas used for calculating motion features for surgical team

**Motion features**	**Formula**	**Description**
Displacement	$displacement_{i,j} = \sqrt{(x_{i,j+1} - x_{i,j})^2 + (y_{i,j+1} - y_{i,j})^2}$	where i is the person ID in each team and j is the time index
Displacement Speed	$speed_{i,j} = \frac{displacement_{i,j}}{\Delta t}$	where is the time between frames, i is the person ID, and j is the time index.
Displacement Speed Variability	$\text{Speed Variability}(j) = \sqrt{\frac{1}{n}\sum_{i=1}^{n}(\text{Speed}(i,j) - \text{Average Spe}}$ [illegible]	Where Speed Variability(j) represents the speed variability of the team at time index j. Speed(i, j) denotes the speed of the person i at time index j. Average Speed(j) is the average speed for the team at time index j. n is the number of team members contributing to the calculation.
Displacement Acceleration	$acceleration_{i,j} = \frac{speed_{i,j} - speed_{i,j-1}}{\Delta t}$	Here, Δt is the time between frames, i is the team person ID, and j is the time index.
Displacement Entropy	$H = -\sum_{i=1}^{n} p_i \log_2 p_i$	The entropy of a given window is calculated using the formula, where p_i is the proportion of observations in the window that belong to symbol i.

© 2024 Harari RE et al. *JAMA Network Open.*

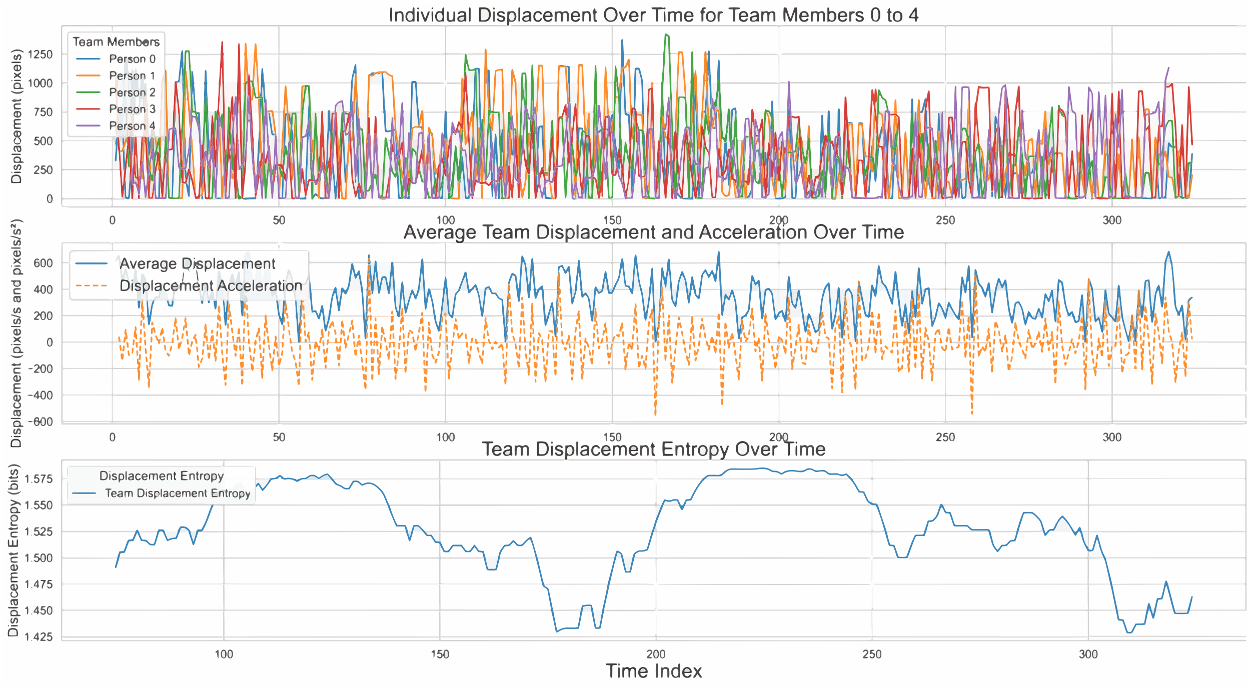

eFigure 1. Movement visualization of surgical team members. First row plots the line chart of individual displacement over time. Second row plots the team's average displacement and acceleration over time. Third row shows team displacement entropy measured over time.

© 2024 Harari RE et al. *JAMA Network Open.*

eFigure 2. Correlation Between Team Motion Features and NOTSS Scores

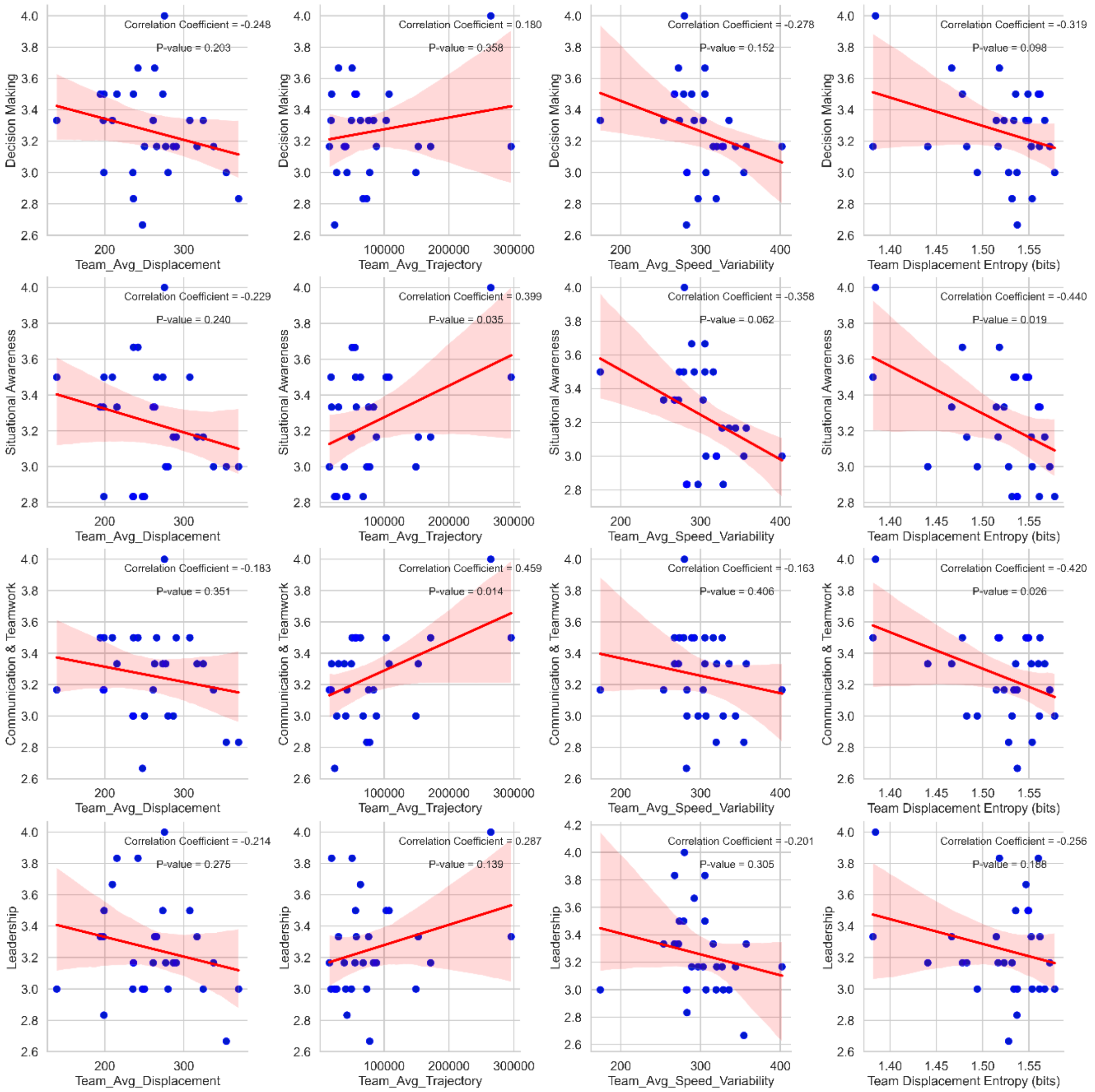

© 2024 Harari RE et al. *JAMA Network Open.*

eTable 2. Linear regression models adjusted for preoperative and intraoperative variables.

Models	Adjusted R^2	F-statistic	p-value	Coefficient	95% CI Lower	95% CI Upper
**Avg. Team Trajectory Model**	.335	5.528				
Constant			.002	9.603e-06	4e-06	1.52e-05
Avg. Team Trajectory			.004	10.51	8.812	12.21
Bypass length			.081	0.0001	-1.92e-05	0.000
30 Day morbidity			.116	9.04	-2.40	20.49
**Avg. Team Displacement Acceleration Model**	.261	2.824				
Constant			.06	12.33	10.44	14.21
Avg. Team Displacement Acceleration			.03	0.370	0.036	0.70
Bypass length			.46	6.975e-05	-0.000	0.000
30 Day morbidity			.61	3.184	-9.64	16.01
**Avg. Team Displacement Entropy Model**	.304	4.928				
Constant			.008	30.79	18.69	42.88
Team Displacement Entropy (bits)			.003	-12.64	-20.54	-4.74
Bypass length			.22	0.0001	-6.58e-05	0.000
30 Day morbidity			.11	9.32	-2.43	21.09
