# Supplementary material for: Deep Learning Analysis of Surgical Video Recordings to Assess Nontechnical Skills
Source: JAMA Netw Open. 2024 Jul 31;7(7):e2422520. doi: 10.1001/jamanetworkopen.2024.22520 (PMC11292454; doi:10.1001/jamanetworkopen.2024.22520)
Supplement: Supplement 2. — Data Sharing Statement [file jamanetwopen-e2422520-s002.pdf]

Data Sharing Statement

Harari. Deep Learning Analysis of Surgical Video Recordings to Assess Nontechnical Skills. *JAMA Netw Open*. Published July 31, 2024. doi:10.1001/jamanetworkopen.2024.22520

Data
Data available: Yes
Data types: Deidentified participant data, Data dictionary
How to access data: https://scholar.harvard.edu/zenati/home
When available: beginning date: 12-01-2025, end date: 12-01-2030

Supporting Documents
Document types: Statistical/analytic code
How to access documents: https://scholar.harvard.edu/zenati/home
When available: beginning date: 12-01-2025, end date: 12-01-2030

Additional Information
Who can access the data: Researchers whose proposed use of the data has been approved.
Types of analyses: any purpose
Mechanisms of data availability: investigator support
